# Angiotensin-converting enzyme 2 overexpression protects against doxorubicin-induced cardiomyopathy by multiple mechanisms in rats

**DOI:** 10.18632/oncotarget.15595

**Published:** 2017-02-21

**Authors:** Hui Ma, Jing Kong, Yu-Lin Wang, Jun-Long Li, Nai-Hao Hei, Xin-Ran Cao, Jing-Jing Yang, Wen-Jiang Yan, Wen-Jing Liang, Hong-Yan Dai, Bo Dong

**Affiliations:** ^1^ Department of Pediatrics, Shandong Provincial Hospital Affiliated to Shandong University, Jinan, China; ^2^ Department of Cardiology, Shandong Provincial Hospital Affiliated to Shandong University, Jinan, China; ^3^ Key Laboratory of Cardiovascular Remodeling and Function Research, Qilu Hospital, Shandong University, Jinan, China; ^4^ Cardiovascular department, Qingdao Municipal Hospital, Qingdao, China

**Keywords:** angiotensin-converting enzyme 2, cilazapril, doxorubicin-induced cardiomyopathy, gene therapy

## Abstract

Angiotensin-converting enzyme 2 (ACE2) is considered a potential therapeutic target of the renin-angiotensin system (RAS) for the treatment of cardiovascular diseases. We aimed to explore the effects of ACE2 overexpression on doxorubicin-induced cardiomyopathy in rats. Rats were randomly divided into treatment and control groups. The rats of treatment group were injected intraperitoneally with 6 doses of doxorubicin (2.5 mg/kg) within a period of two weeks. Two weeks after the initial injection of doxorubicin, these rats were randomly divided into Mock, Ad-EGFP, Ad-ACE2, and Cilazapril groups. The rats of Ad-EGFP and Ad-ACE2 groups received intramyocardial injection of Ad-EGFP and Ad-ACE2, respectively. The rats of Cilazapril group received cilazapril (10 mg/kg/day) via intragastric intubation. Apoptosis, inflammation, oxidative stress, cardiac function, the extent of myocardial fibrosis, and levels of ACE2, ACE, angiotensin II (AngII), and angiotensin (1–7) were evaluated. Four weeks after ACE2 gene transfer, the Ad-ACE2 group showed not only reduced apoptosis, inflammatory response, oxidative stress, left ventricular (LV) volume, extent of myocardial fibrosis and mortality of rats, but also increased LV ejection fraction and ACE2 expression level compared with the Mock and Ad-EGFP groups. ACE2 overexpression was superior to cilazapril in improving doxorubicin-induced cardiomyopathy. The putative mechanisms may involve activation of the AMPK and PI3K-AKT pathways, inhibition of the ERK pathway, decrease of TGF-β1 expression, and interactions of shifting RAS components, such as decreased myocardium AngII levels, increased myocardium Ang (1–7) levels, and reduced ACE expression. Thus, ACE2 may be a novel therapeutic approach to prevent and treat doxorubicin-induced cardiomyopathy.

## INTRODUCTION

Doxorubicin is a commonly used chemotherapeutic agent for the treatment of solid and haematologic tumors. However, its cardiotoxic side effects and the development of severe congestive heart failure limit the application of this potent chemotherapeutic agent [1, 2]. The mechanisms of doxorubicin-induced cardiomyopathy remain not fully elucidated. Pathologically, doxorubicin-induced cardiomyopathy was associated with heightened oxidative stress status, apoptosis of cardiac cells, inflammatory response, myocardial fibrosis, metabolism abnormality, DNA damage and mitochondrial damage [3–7]. Several agents including anti-oxidants, angiotensin-converting enzyme inhibitors (ACEI), angiotensin receptor antagonists (ARB) and dexrazoxan have been employed for the attenuation of doxorubicin-induced cardiomyopathic damage [8, 9]. Unfortunately, all of these agents have not yet gained effective enough evidence to justify a routine use. Hence, the search for a safe and effective therapeutic method of preventing doxorubicin-induced cardiomyopathy remains an important issue in both cardiology and oncology.

The renin-angiotensin system (RAS) plays an important role in the pathophysiology of doxorubicin-induced cardiomyopathy, and the inhibition of ACE/AngII/AngII type 1 (AT1) receptor (AT1R) axis has been shown to improve doxorubicin-induced cardiomyopathy [8, 10, 11]. Traditionally, ACEI and ARB are involved in inhibiting the synthesis of AngII and preventing activation of AT1R. However, the degradation of AngII may also function as regulating AngII levels, especially at a tissue level. ACE2 is considered a potential therapeutic target of RAS for the treatment of cardiovascular diseases due to its key role in limiting the vasoconstrictor action of AngII through its inactivation, and counteracting the actions of AngII through the formation of Ang (1–7), which is reported to have prevented left ventricular fibrosis and dysfunction at the Ang (1–7) or mas receptor [12]. Crackower et al. first reported a severe myocardial contractile dysfunction in ACE2-knockout mice [13]. Our previous study found that ACE2 overexpression attenuated myocardial collagen accumulation and improved left ventricular remodeling and function in a rat model of myocardial infarction [14]. Other studies also reported that ACE2/Ang (1–7) axis played a role in cardiac physiology and in the pathophysiology of heart failure [15].

ACE2, a homologue of ACE, catalyzes conversion of AngI to the inactive nonapeptide Ang (1–9) and conversion of AngII to a vasodilative heptapeptide Ang (1–7), thereby functioning effectively as an endogenous ACE inhibitor [16]. We took the approach of direct injection of ACE2 adenoviral vectors into heart in the current study. Although intramyocardial injection of ACE2 adenoviral vectors may induce myocardial damage when compared with oral drugs [17], little study has been reported about the direct effects of ACE2 gene overexpression on doxorubicin-induced cardiomyopathy. In this study, we hypothesized that ACE2 overexpression may improve cardiac function and decrease mortality of rats with doxorubicin-induced cardiomyopathy, and that ACE2 is superior to ACEI in the treatment of doxorubicin-induced cardiomyopathy. Based on this hypothesis, we determined the effects of ACE2 overexpression via an adenovirus vector or ACEI on doxorubicin-induced cardiomyopathy, including cell apoptosis, inflammatory response, oxidative stress, and myocardial fibrosis.

## RESULTS

### General observations and mortality

The general health of the rats was monitored as well as mortality prior to sacrifice during a period of 4 weeks after gene treatment. We found that rats in the Mock and Ad-EGFP groups developed scruffy, light yellowish fur, diarrhea and red exudates around the eyes with grossly enlarged abdomen and ascites. In contrast, rats in the Ad-ACE2 group showed no diarrhea, eye discharge, or ascites, while rats in the Cilazapril group had slight symptoms above.

**Figure 1:**
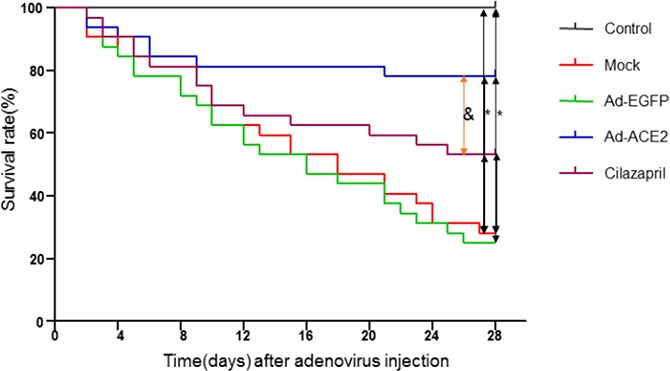
Kaplan-Meier survival curves of five groups of rats after adenovirus injection The effect of treatment with Ad-ACE2 or cilazapril on mortality in rats with doxorubicin-induced cardiomyopathy. Survival rate was monitored over a 4-week period following initial adenovirus myocardial injection as compared with Mock and Ad-EGFP groups. *P < 0.05 vs. Control, Ad-ACE2 and Cilazapril. ^&^*P* < 0.05 vs. Ad-ACE2.

Of the 170 animals in the treatment group, 10 rats died during the initial 2 weeks of doxorubicin injection and were not included in the survival calculations, as it is considered that mortality at this stage was not a result of cardiac dysfunction, but rather related to peritonitis or toxic systemic effects [18]. As shown in Figure 1, 23 of Mock group rats, 24 of Ad-EGFP group rats, 6 of Ad-ACE2 group rats and 15 of Cilazapril group rats died in the 4-week period following the completion of the treatment with doxorubicin, yielding a cumulative mortality of 71.88%, 75.00%, 18.75% and 46.88%, respectively. No death was found in the control group. The mortality of rats in Ad-ACE2 and Cilazapril groups was significantly lower than that in the Mock and Ad-EGFP groups. In contrast, the mortality of rats in the Ad-ACE2 group was lower than that in the Cilazapril group.

### Efficiency of ACE2 gene transfer

**Figure 2:**
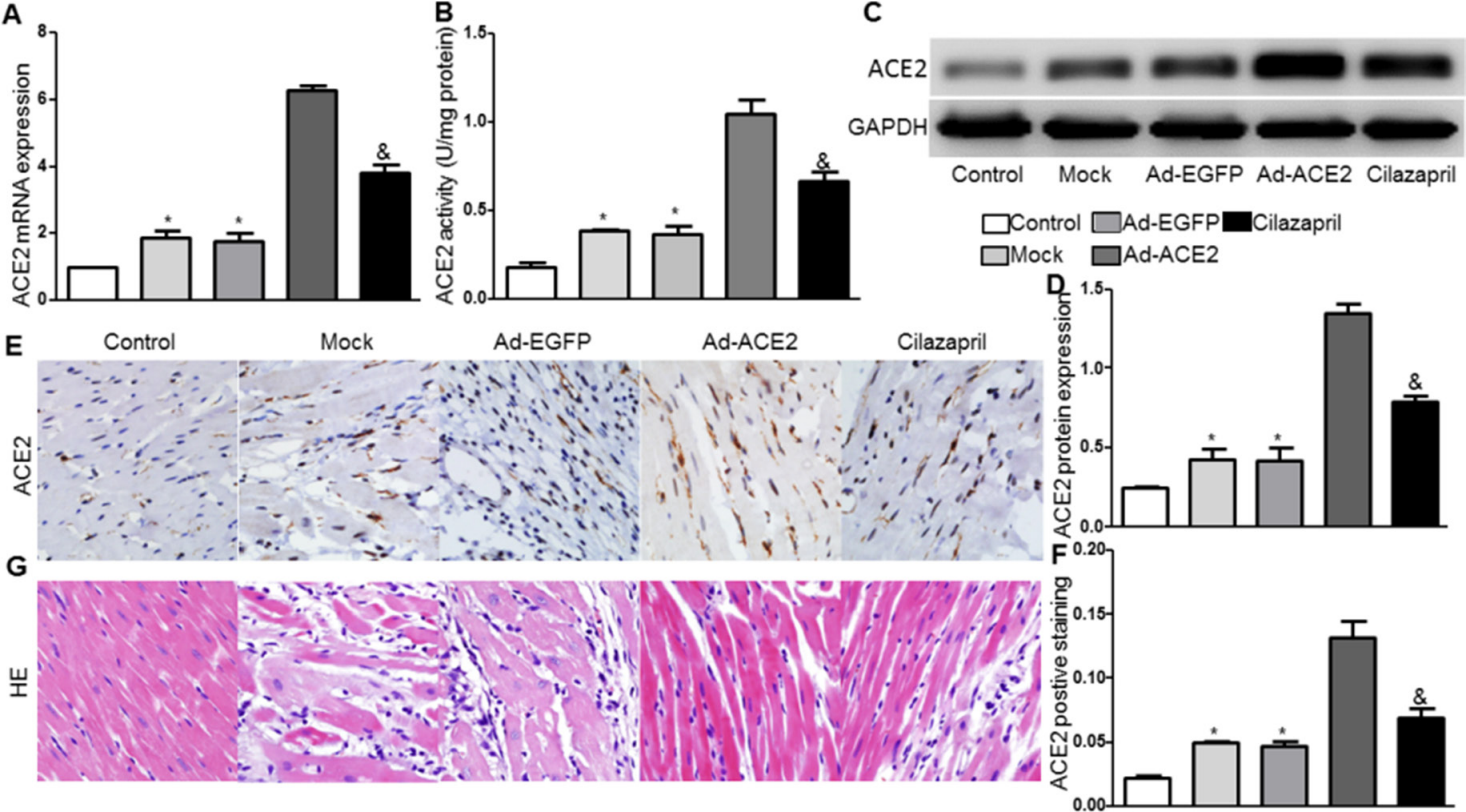
ACE2 expression and activity in 5 groups of rats after gene transfer (**A**) ACE2 messenger ribonucleic acid (mRNA) expression was assayed by real time PCR in 5 groups of rats 2 weeks after gene transfer. (**B**) ACE2 activity. (**C**) Western blot analysis of the protein levels of ACE2 in homogenates of myocardium from five groups 2 weeks after gene transfer and (**D**) quantitative analysis of ACE2 protein expression in C. The blot is a representative of three blots from three independent experiments. (**E**) Immunohistochemistry (IHC) analysis of ACE2 of myocardium cross-section from each group rat 2 weeks after gene transfer and (**F**) quantitative analysis of ACE2 positive staining in E. Scale bar: 20 μm. *P < 0.05 vs. Control, Ad-ACE2 and Cilazapril. ^&^*P* < 0.05 vs. Ad-ACE2. (**G**) Representative hematoxylin and eosin staining of myocardium cross-section in the five groups of rats 4 weeks after gene transfer. Scale bar: 20 μm. N is 8–15 in each group.

Compared with the Mock and Ad-EGFP groups, the mRNA and protein expression levels and the activity of ACE2 were significantly higher in the Ad-ACE2 group 2 weeks after ACE2 transfection, with a slight increase in the Cilazapril group. And these measurements was significantly lower in the Cilazapril group than in the Ad-ACE2 group (Figure 2A–2F). The results also showed that ACE2 mRNA and protein expressions and activity were increased in the Mock and Ad-EGFP groups compared with the control group.

### Pathological changes

Analysis of H&E stained sections in the Mock and Ad-EGFP groups of rats were characterized by inflammatory cells invasion, loss of myofibrils and disorganization. However, these pathological changes were alleviated in sections from Ad-ACE2 and Cilazapril groups, and the alleviative degree of the pathological changes was less in the Cilazapril group than in the Ad-ACE2 group (Figure 2G).

### TUNEL assay and AMPK, caspase3 and Bcl-2 protein expression

**Figure 3:**
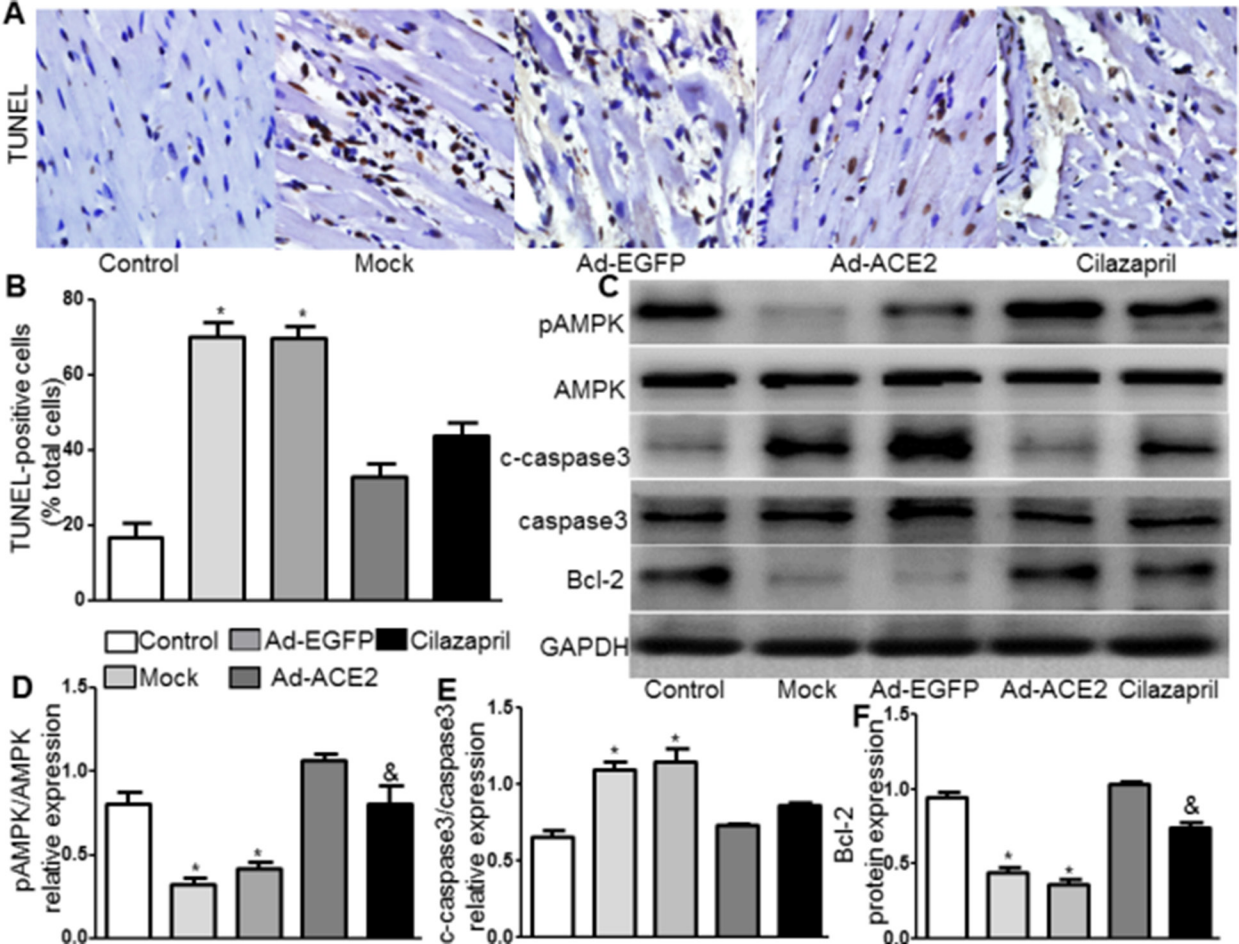
TUNEL staining and AMPK, caspase3, and Bcl-2 protein expression in five groups of rats 4 weeks after gene transfer (**A**) Apoptosis is detected by TUNEL staining in myocardial tissue sections from each group rat 4 weeks after gene transfer. Representative TUNEL staining images are shown. scale bar: 20 μm. (**B**) Quantitative analysis of TUNEL-positive staining cells. (**C**) The levels of pAMPK, AMPK, c-caspase3, caspase3 and Bcl-2 protein in myocardium were determined by Western blot and (**D**–**F**) quantitative analysis of pAMPK/AMPK, c-caspase3/caspase3 and Bcl-2 protein in C. The blot is a representative of three blots from three independent experiments. N is 8–15 in each group. *P < 0.05 vs. Control, Ad-ACE2 and Cilazapril. ^&^*P* < 0.05 vs. Ad-ACE2.

TUNEL assay was performed and the results showed that radio of TUNEL positive cells were significantly decreased in the Ad-ACE2 and Cilazapril groups compared with the Mock and Ad-EGFP groups, with no significant difference between the Mock and Ad-EGFP groups or between the Ad-ACE2 and Cilazapril groups (Figure 3A and 3B). The pAMPK(Thr172)/AMPK radio in myocardium was significantly higher in the Ad-ACE2 and Cilazapril groups than in the Mock and Ad-EGFP groups. However, this radio was significantly lower in the Cilazapril group than in the Ad-ACE2 group (Figure 3C and 3D). The c-caspase3/caspase3 radio in myocardium was also significantly decreased in the Ad-ACE2 and Cilazapril groups compared with the Mock and Ad-EGFP groups, with no significant difference between the Mock and Ad-EGFP groups or between the Ad-ACE2 and Cilazapril groups (Figure 3C and 3E). In contrast, Bcl-2 protein expression by western blot analysis was significantly increased in the Ad-ACE2 and Cilazapril groups compared with the Mock and Ad-EGFP groups, and was significantly decreased in the Cilazapril group compared with the Ad-ACE2 group (Figure 3C and 3F).

### Expression levels of inflammatory cytokines

**Figure 4:**
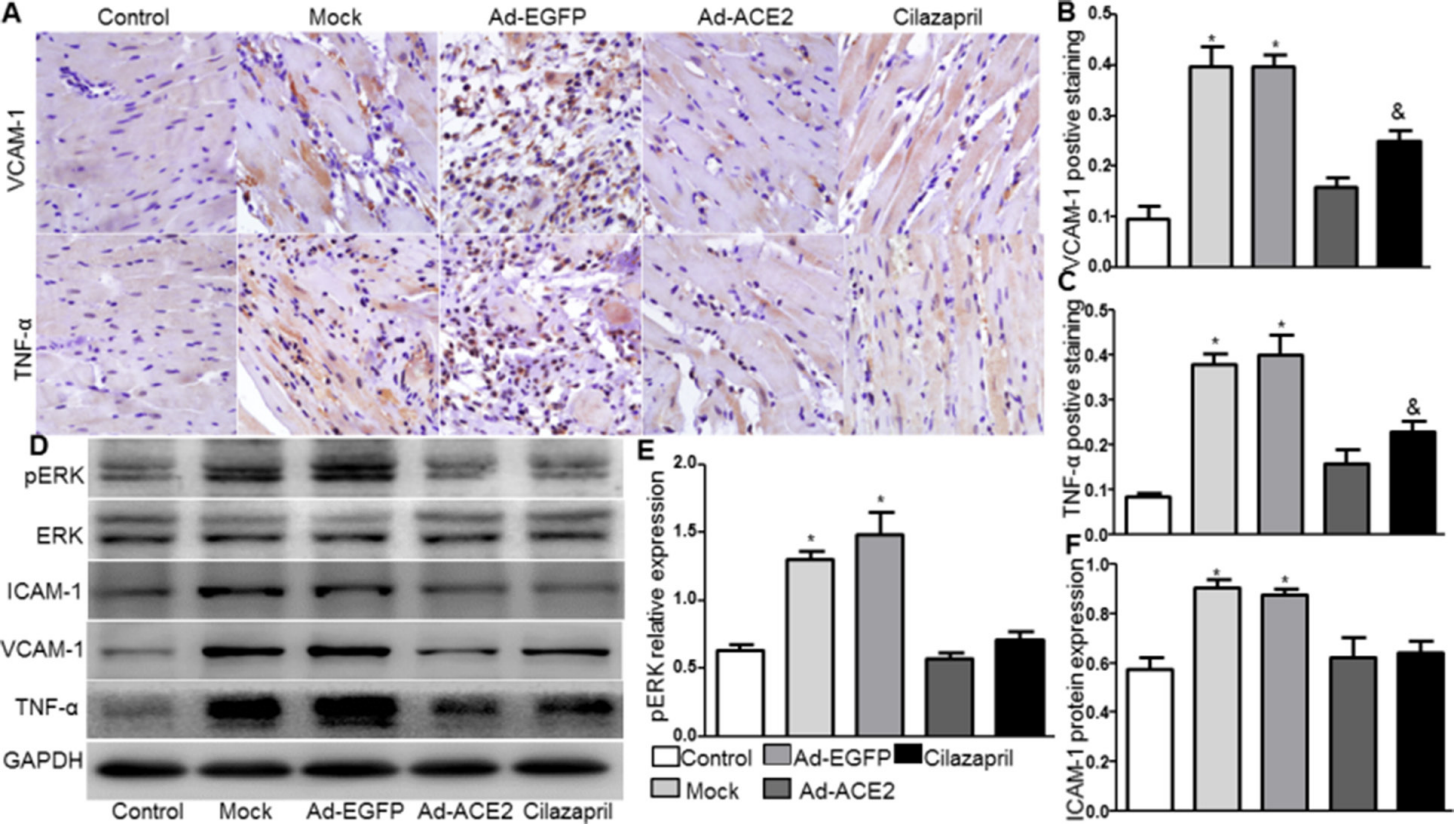
VCAM-1, TNF-α, ICAM-1 and ERK protein expression in five groups of rats 4 weeks after gene transfer (**A**) IHC analysis of VCAM-1 and TNF-α of myocardium cross-section from each group and (**B** and **C**) quantitative analysis of the results in A. Scale bar: 20 μm. (**D**) The levels of ERK, ICAM-1, VCAM-1 and TNF-α protein in myocardium were determined by Western blot and (**E** and **F**) quantitative analysis of ERK and ICAM-1 protein in D. The blot is a representative of three blots from three independent experiments. N is 8–15 in each group. *P < 0.05 vs. Control, Ad-ACE2 and Cilazapril;^&^*P* < 0.05 vs. Ad-ACE2.

The protein expression levels of VCAM-1 and TNF-α by immunohistochemical analysis in Ad-ACE2 and Cilazapril groups were significantly lower than those in the Mock and Ad-EGFP groups. However, the VCAM-1 and TNF-α protein expression in the Ad-ACE2 group was lower than in the Cilazapril group (Figure 4A–4C). Immunohistochemical results were further confirmed by western blot analysis, a similar distribution in all 5 groups (Figure 4D). Similarly, the protein expression level of ICAM-1 by western blot analysis in Ad-ACE2 and Cilazapril groups was significantly lower than in the Mock and Ad-EGFP groups, with no statistical difference between the Mock and Ad-EGFP groups or between the Ad-ACE2 and Cilazapril groups (Figure 4D and 4F).

### ERK protein expression

Compared with Mock and Ad-EGFP groups, phosphorylated ERK protein expression by western blot analysis was significantly reduced in the Ad-ACE2 and Cilazapril groups (Figure 4D and 4E), with no significant difference between the Mock and Ad-EGFP groups or between the Ad-ACE2 and Cilazapril groups.

### NOX2, P47 and iNOS protein expression and SOD activity

**Figure 5:**
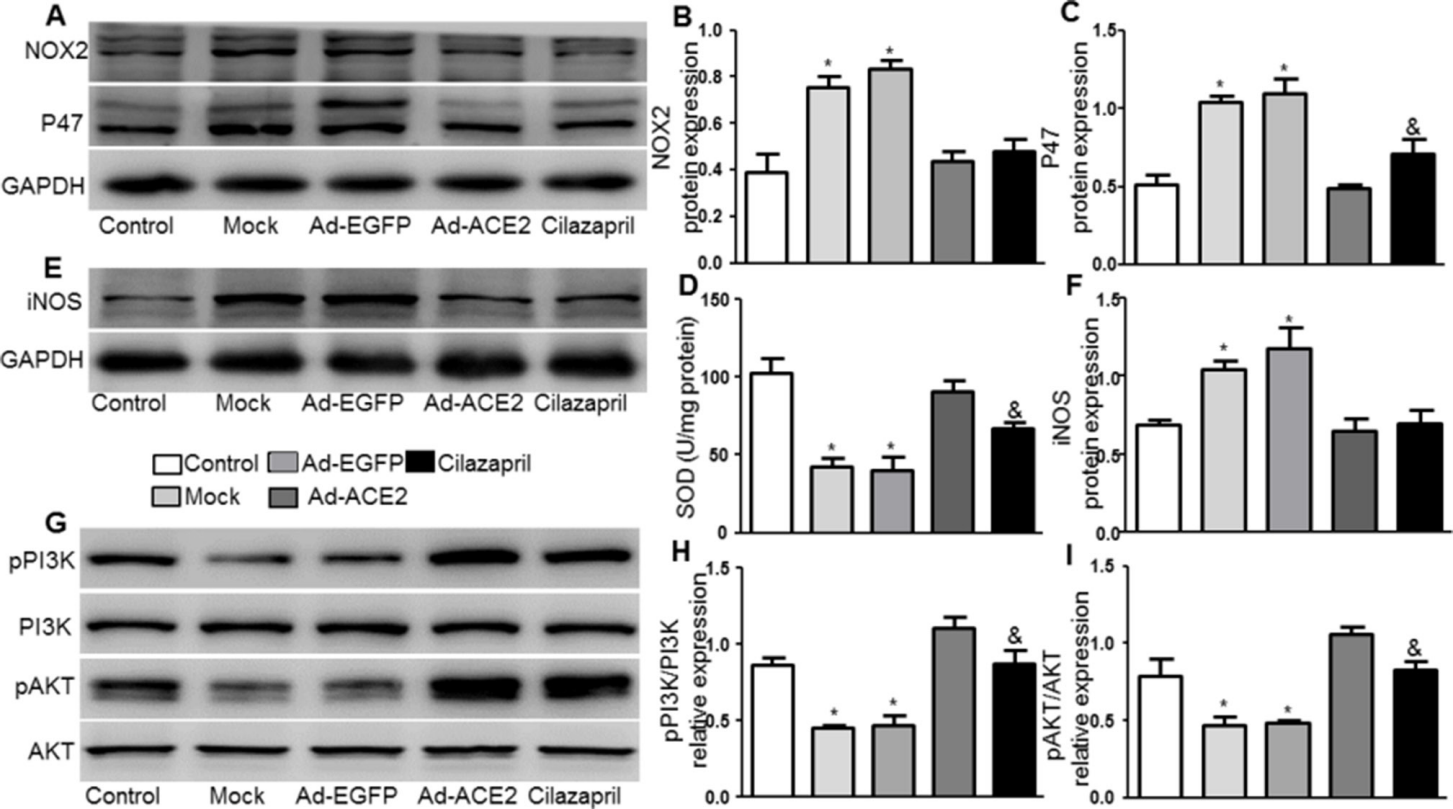
NOX2, P47, iNOS, PI3K, and AKT protein expression and SOD activity in five groups of rats 4 weeks after gene transfer (**A**) The levels of NOX2 and P47 protein in myocardium were determined by Western blot and (**B** and **C**) quantitative analysis of NOX2 and P47 protein in A. (**D**) The levels of SOD activity in myocardium. (**E**) Western blotting analysis of the protein levels of iNOS in homogenates of myocardium from five group rats and (**F**) quantitative analysis of iNOS protein level. (**G**) The levels of pPI3K, PI3K, pAKT and AKT protein in myocardium were determined by Western blot and (**H** and **I**) quantitative analysis of pPI3K/PI3K, pAKT /AKT in F. The blot is a representative of three blots from three independent experiments. N is 8–15 in each group. *P < 0.05 vs. Control, Ad-ACE2and Cilazapril; ^&^*P* < 0.05 vs. Ad-ACE2.

The protein expression levels of NOX2,P47 by western blot analysis were substantially lower in the Ad-ACE2 and Cilazapril groups than in the Mock and Ad-EGFP groups. Furthermore, the P47 protein expression in the Cilazapril group was higher than in the Ad-ACE2 group, while the protein expression level of NOX2 had no statistically difference between the Ad-ACE2 and Cilazapril groups (Figure 5A–5C). Additionally, SOD activity was significantly increased in the Ad-ACE2 and Cilazapril groups compared with the Mock and Ad-EGFP groups (Figure 5D). However, SOD activity in the Ad-ACE2 group was higher than in the Cilazapril group. As shown in Figure 5E and 5F, in the Mock and Ad-EGFP groups, iNOS protein expression by western blot analysis was significantly increased compared to Ad-ACE2 and Cilazapril groups, with no statistical difference between the Mock and Ad-EGFP groups or between the Ad-ACE2 group and Cilazapril group.

### Expression levels of PI3K and AKT

The pPI3K /PI3K and pAKT/AKT radios in myocardium were significantly lower in the Mock and Ad-EGFP groups than in the control, Ad-ACE2 and Cilazapril groups (Figure 5G–5I). In addition, these results in the Ad-ACE2 group were significantly increased compared with the Cilazapril group. There was no statistical difference between the Mock and Ad-EGFP groups.

### Echocardiographic measurements

**Figure 6:**
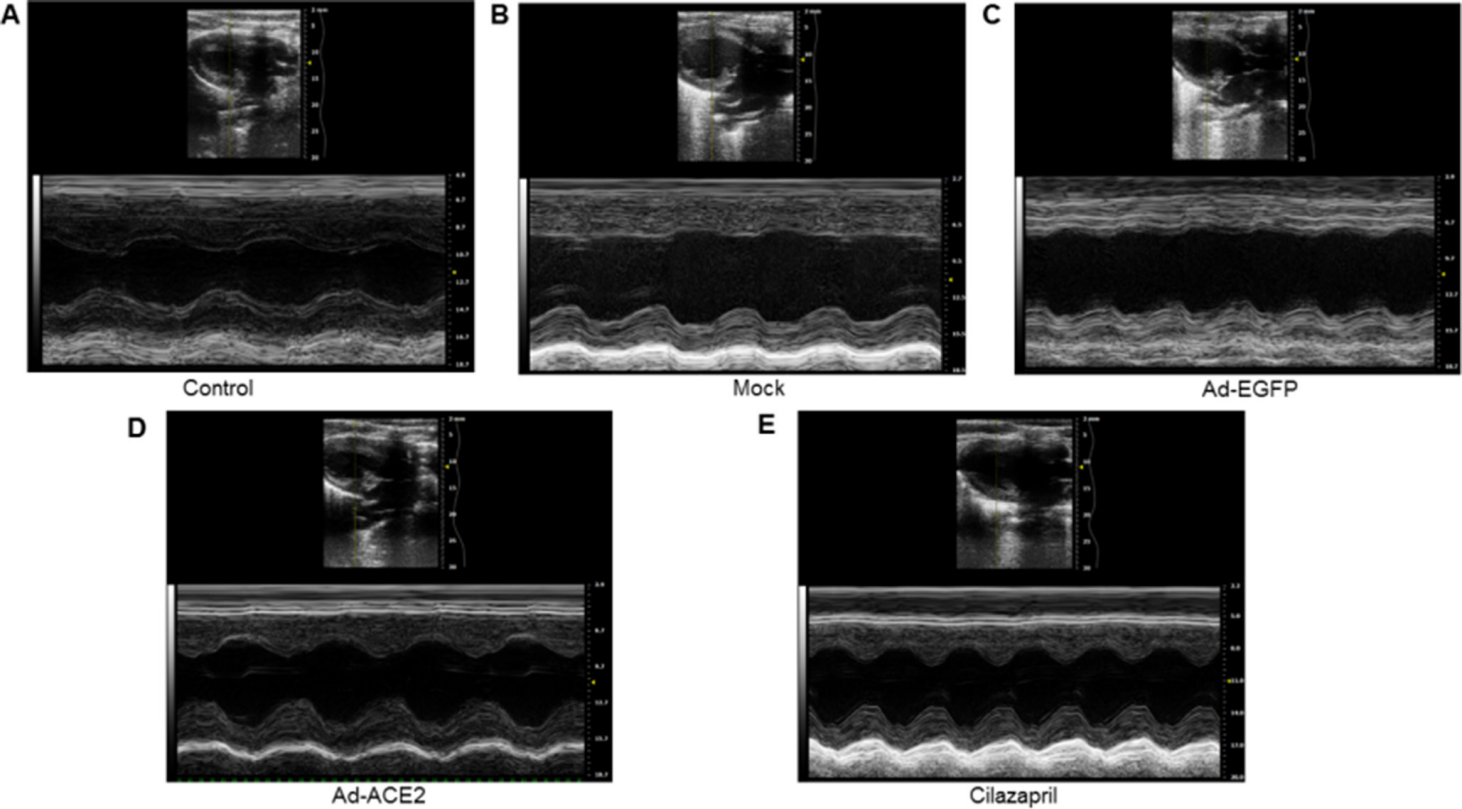
Cardiac function data in five groups of rats 4 weeks after gene transfer Representative echocardiographic graphs in 2D parasternal long axis view and M-mode of the left ventricle (LV) at the level of the papillary muscles. N is 8–15 in each group.

As shown in Figure 6 and Table 1, LV end-systolic diameter and LVEDD were significantly decreased, whereas LVEF and FS were increased in the Ad-ACE2 and Cilazapril groups in comparison with the Mock and Ad-EGFP groups. In addition, LVEDD were higher, whereas LVEF and FS were lower in the Cilazapril group than in the Ad-ACE2 group. However, ACE2 and cilazapril had no significant effect on blood pressure and heart rate in doxorubicin-induced cardiomyopathy rats (Table 1).

**Table 1:** Echocardiographic measurements in 5 groups of rats 4 weeks after gene transfer

Groups	Control	Mock	Ad-EGFP	Ad-ACE2	Cilazapril
HR (bpm)	366.78 ± 12.93	372.38 ± 20.72	362.19 ± 18.02	377.53 ± 11.28	356.28 ± 17.28
sBP (mmHg)	124.73 ± 6.24	80.26 ± 3.27^#^	77.28 ± 4.83^#^	83.05 ± 5.50^#^	84.37 ± 6.98^#^
dBP (mmHg)	96.08 ± 5.32	62.83 ± 3.37^#^	57.37 ± 2.94^#^	65.27 ± 4.21^#^	63.89 ± 3.19^#^
LVEF (%)	72.34 ± 0.84	38.86 ± 2.22*	35.81 ± 2.24*	60.64 ± 0.79	50.5 ± 1.65^&^
ES (%)	42.31 ± 0.49	19.48 ± 1.38*	17.77 ± 1.24*	36.24 ± 0.44	28.61 ± 1.25^&^
LVESD (mm)	3.12 ± 0.27	5.52 ± 0.16*	5.68 ± 0.57*	4.11 ± 0.07	4.24 ± 0.17
LVEDD (mm)	4.90 ± 0.51	7.53 ± 0.26*	7.66 ± 0.19*	6.08 ± 0.36	6.79 ± 0.26^&^

HR, heart rates; sBP, systolic blood pressure; dBP, diastolic blood pressure. LVEF, left ventricular ejection fraction; ES, left ventricular fractional shortening; LVESD, left ventricular end-systolic diameter; LVEDD, left ventricular end-diastolic diameter. N is 8–15 in each group. Data were expressed by mean ± s.e.m. ^#^*P* < 0.05 vs. Control; *P < 0.05 vs. Control, Ad-ACE2 and Cilazapril; ^&^*P* < 0.05 vs. Ad-ACE2.

### Expression levels of collagen

**Figure 7:**
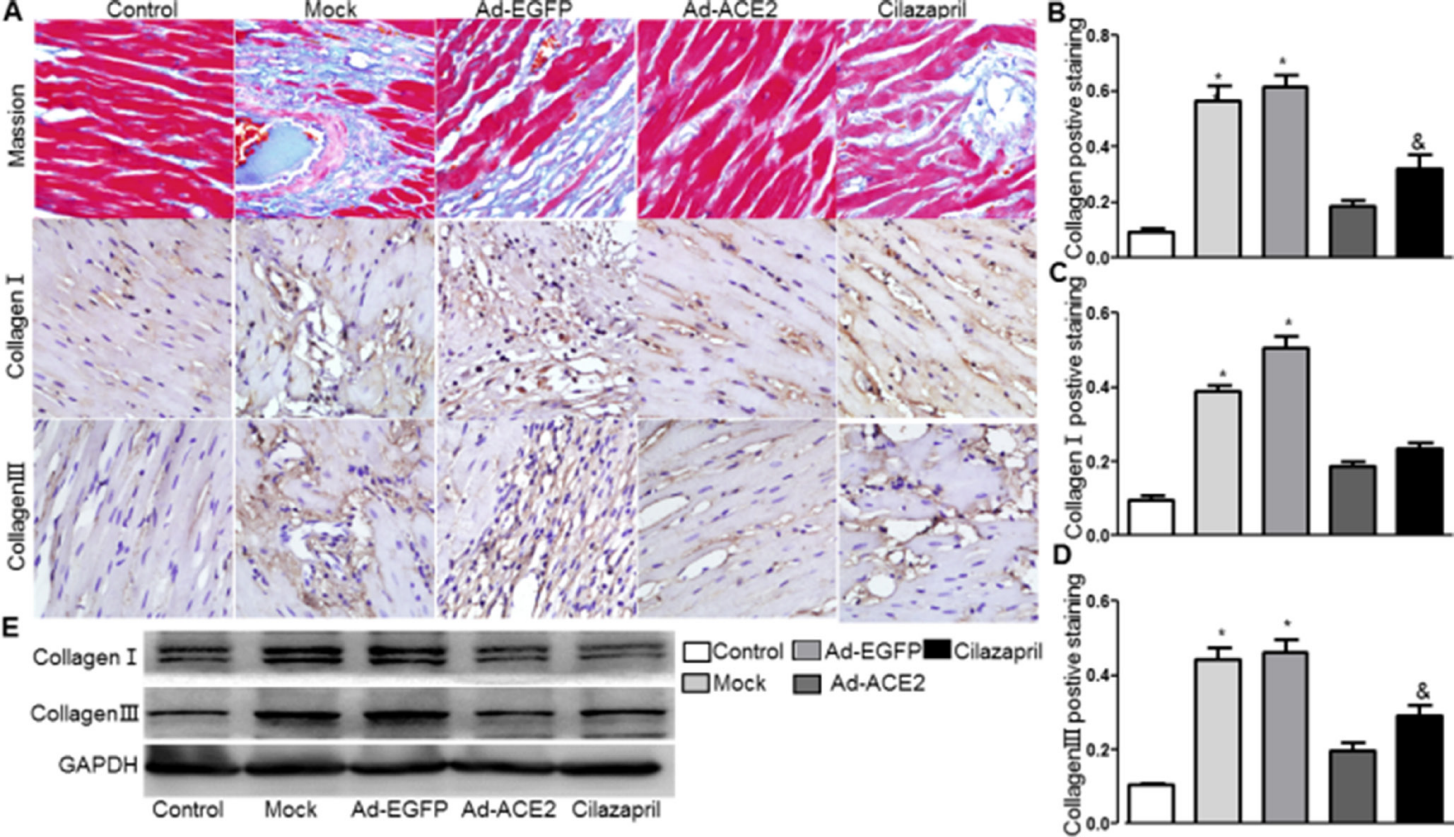
Masson's trichrome staining of myocardium and collagen protein expression in five groups of rats 4 weeks after gene transfer (**A**) Representative Masson's trichrome staining, and IHC analysis of collagen I and collagen III of myocardium cross-section from each group. Scale bar: 20 μm. (**B**–**D**) quantitative analysis of collagen, collagen I and collagen III in A. (**E**) Western Blotting analysis of the protein levels of collagen I and collagen III in homogenates of myocardium from five group rats. The blot is a representative of three blots from three independent experiments. N is 8–15 in each group. *P < 0.05 vs. Control, Ad-ACE2 and Cilazapril; ^&^*P* < 0.05 vs. Ad-ACE2.

The result showed that the cardiac collagen deposition in Ad-ACE2 and Cilazapril groups was significantly lower than in the Ad-EGFP and Mock groups. Furthermore, the measurement in Ad-ACE2 group was significantly lower than in Cilazapril group. There was no statistical difference between the Ad-EGFP group and Mock group (Figure 7A and 7B). The protein expression levels of collagen I and III by immunohistochemical analysis in Ad-ACE2 and Cilazapril groups were significantly decreased compared with the Mock and Ad-EGFP groups. Furthermore, the collagen I protein expression in the Ad-ACE2 group was lower than in the Cilazapril group, while the protein expression level of collagen III had no statistically difference between the Ad-ACE2 and Cilazapril groups (Figure 7A, 7C and 7D). Immunohistochemical results were further verified by western blot analysis, a similar distribution in all 5 groups (Figure 7E).

### TGF-β1, MMP-9, MMP-1, TIMP-1 and TIMP-2 protein expression

**Figure 8:**
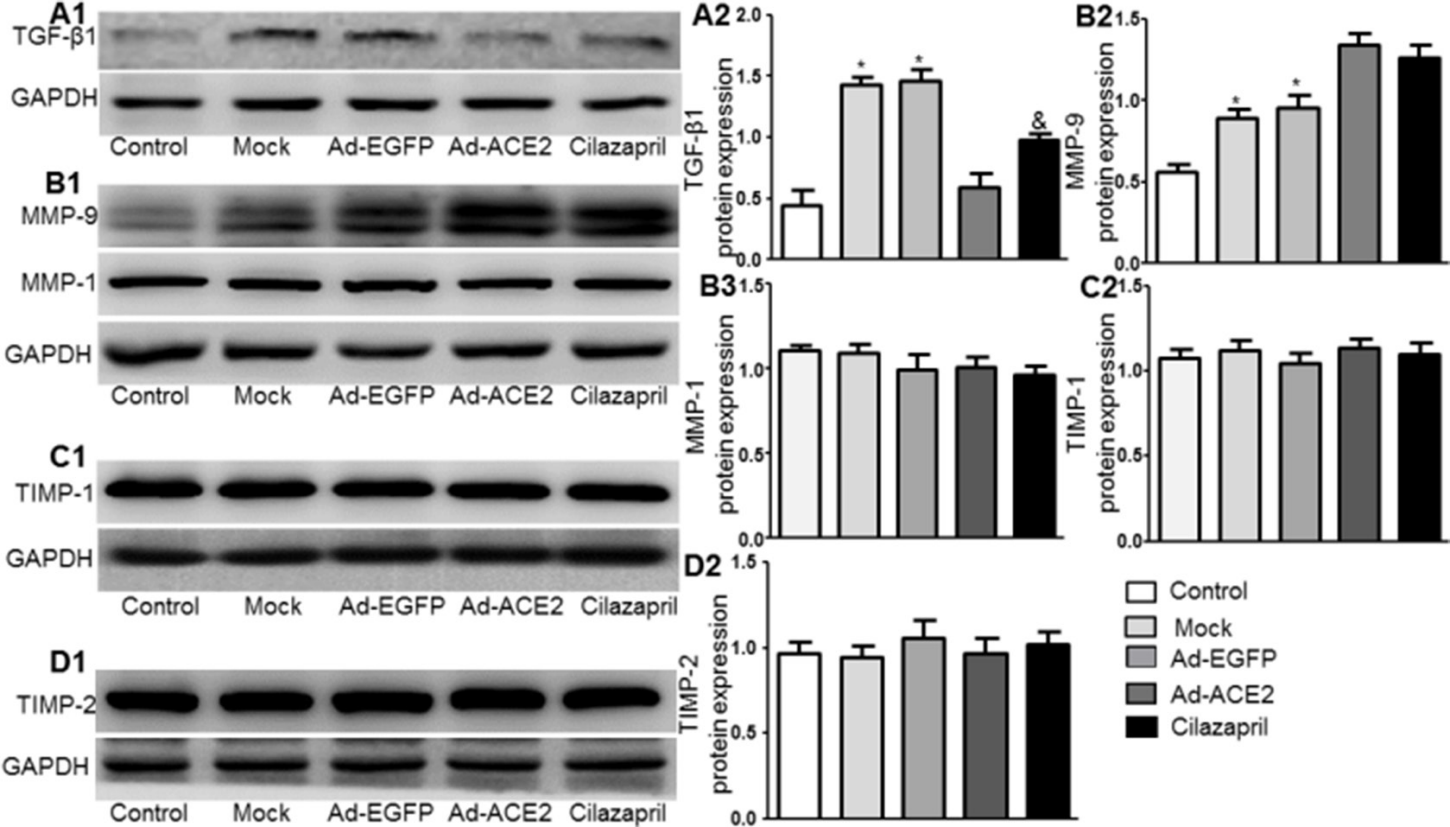
TGF-β1, MMP-9, MMP-1, TIMP-1 and TIMP-2 protein expression in five groups of rats 4 weeks after gene transfer (**A**1–**D**1) Western blotting analysis of the protein levels of TGF-β1, MMP-9, MMP-1, TIMP-1 and TIMP-2 in homogenates of myocardium from five group rats and (A2–D2 and B3) quantitative analysis of TGF-β1, MMP-9, MMP-1, TIMP-1 and TIMP-2 protein. The blot is a representative of three blots from three independent experiments. N is 8–15 in each group. *P < 0.05 vs. Control, Ad-ACE2 and Cilazapril; ^&^*P* < 0.05 vs. Ad-ACE2.

The TGF-β1 protein expression in the Ad-ACE2 and Cilazapril groups was significantly lower than in the Mock and Ad-EGFP groups. In addition, the protein expression level of TGF-β1 in the Cilazapril group was higher than in the Ad-ACE2 group, with no statistical difference between the Mock and Ad-EGFP groups (Figure 8A1 and 8A2). Western blot analysis of MMP9 protein expression (Figure 8B1 and 8B2) in the Mock and Ad-EGFP groups were significantly lower than in the Ad-ACE2 and Cilazapril groups and were significantly increased in comparison with the control group, with no statistically difference between the Mock and Ad-EGFP groups or between the Ad-ACE2 and Cilazapril groups. Additionally, the MMP1 (Figure 8B1 and 8B3), TIMP-1 (Figure 8C1 and 8C2) and TIMP-2 (Figure 8D1 and 8D2) protein expression levels were similar among the 5 groups.

### ACE2, ACE, AngII, and Ang (1–7) expression

**Figure 9:**
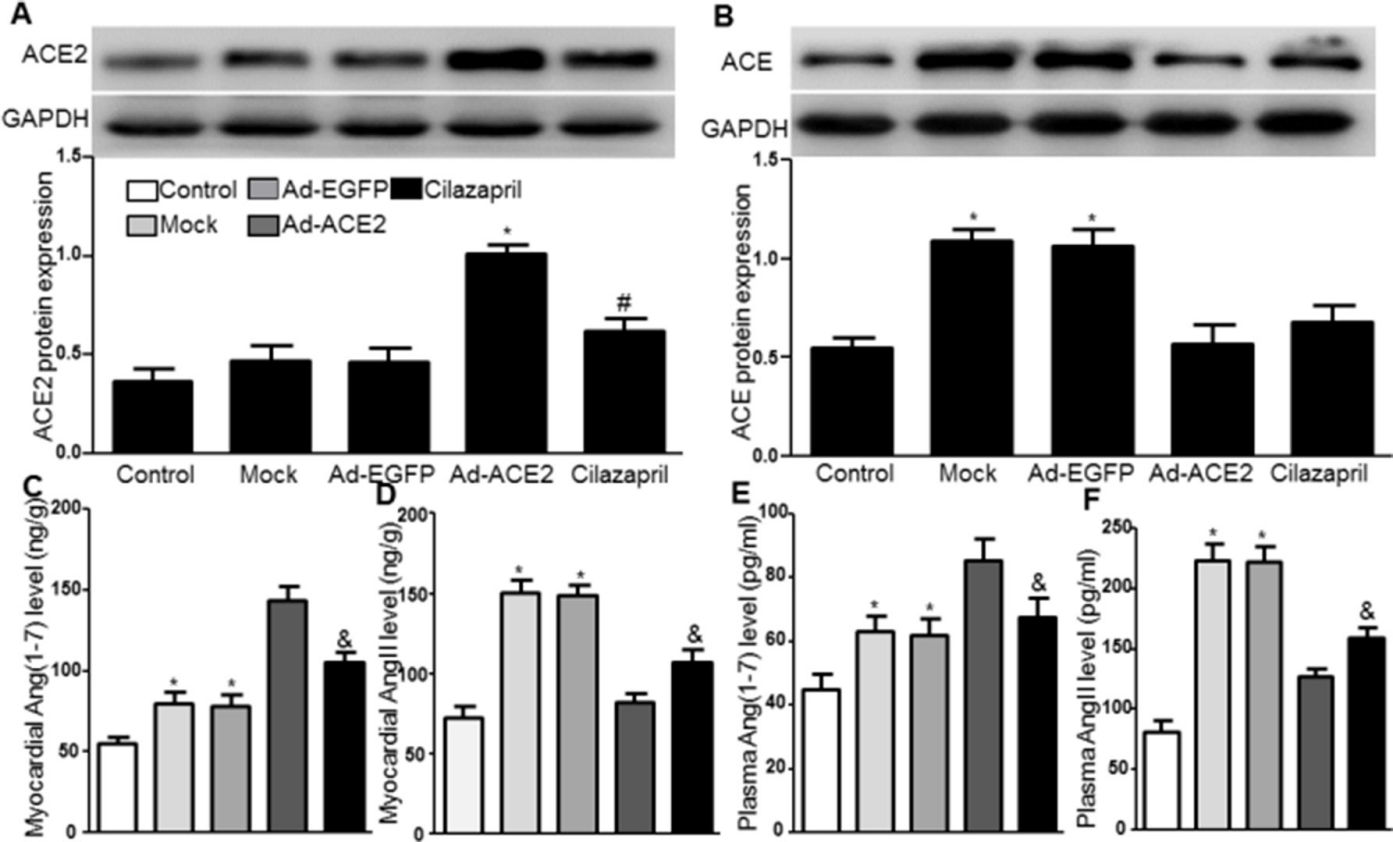
ACE2, ACE protein expression and the levels of AngII and Ang (1–7) in five groups of rats 4 weeks after gene transfer (**A**) The levels of ACE2 protein in myocardium were detected by Western blot 4 weeks after gene transfer and quantitative analysis of ACE2 protein. The blot is a representative of three blots from three independent experiments. 8–15 rats in each group. *P < 0.05 vs. Control, Mock, Ad-EGFP and Cilazapril. ^#^*P* < 0.05 vs. Control. (**B**) Western blotting analysis of the protein levels of ACE in homogenates of myocardium from five group rats and quantitative analysis of ACE protein. *P < 0.05 vs. Control, Ad-ACE2 and Cilazapril. (**C**–**F**) AngII and Ang (1–7) in the myocardium and plasma were detected by ELISA. N is 8–15 in each group. *P < 0.05 vs. Control, Ad-ACE2 and Cilazapril; ^&^*P* < 0.05 vs. Ad-ACE2.

Western blot analysis showed that ACE2 protein expression in the Ad-ACE2 group was significantly increased compared with the control, Mock, Ad-EGFP and Cilazapril groups 4 weeks after gene transfection. In addition, ACE2 expression in the Cilazapril group was slightly increased compared to the control group but not compared to the Mock and Ad-EGFP groups (Figure 9A). In contrast, ACE protein expression was significantly higher in the Mock and Ad-EGFP groups than in the control, Ad-ACE2 and Cilazapril groups, with no statistically difference between the Mock and Ad-EGFP groups or between the Ad-ACE2 and Cilazapril groups (Figure 9B). As revealed by ELISA, myocardial and plasma Ang (1–7) levels showed significantly increase in the Ad-ACE2 group compared with the Mock and Ad-EGFP groups. In contrast, the measurements were only slightly higher in the Cilazapril group than in the Mock and Ad-EGFP groups, and were lower in the Cilazapril group than in the Ad-ACE2 group (Figure 9C and 9E). In contrast, myocardial and plasma AngII protein expression levels by ELISA were lower in the Ad-ACE2, Cilazapril and control groups than in the Mock and Ad-EGFP groups, and these were higher in the Cilazapril group than in the Ad-ACE2 group (Figure 9D and 9F).

## DISCUSSION

There were two key findings of the present study, the first was that ACE2 overexpression decreased apoptosis, inflammatory response, and oxidative stress in doxorubicin-induced cardiomyopathy. The second was that ACE2 overexpression attenuated myocardial fibrosis, improved left ventricular remodeling and cardiac function, and improved pathological symptoms and decreased mortality of rats by Kaplan-Meier survival curves in doxorubicin-induced cardiomyopathy. The mechanisms underlying these effects involved activation of the AMPK and PI3K-AKT pathways, inhibition of the ERK pathway, and decrease of TGF-β1 expression. Furthermore, ACE2 overexpression decreased myocardium AngII levels, increased myocardium Ang (1–7) levels, and reduced ACE expression in the rat model of doxorubicin-induced cardiomyopathy.

Apoptosis was a major cause of cardiac dysfunction in doxorubicin-induced animal models and patients of heart failure [19, 20]. Doxorubicin induced oxidative stress and the abnormal expression of pro-apoptosis protein cleaved caspase3 and anti-apoptosis protein bcl-2, which activated apoptotic signaling resulting in cardiomyocyte apoptosis in the heart [21–23]. High levels of NO production via inducible NO synthase(iNOS) is involved in cardiomyocyte oxidative damage and apoptosis [24]. Our results showed that ACE2 overexpression decreased iNOS expression. Activated NOX2 can generate ROS, which results in cardiomyocyte apoptosis [25]. NOX-produced ROS is regulated through transcriptional repression of the p47phox gene [26]. Previous study also showed that doxorubicin increased the expression of p47phox [27]. Moreover, increasing the activities of antioxidant enzymes, such as SOD, in the heart protect mice against doxorubicin-induced damage [28]. ACE2 overexpression prevented the hypoxia-induced cardiomyocyte apoptosis [29]. In contrast, ACE2 knockout aggravated AngII-induced reactive oxygen species and apoptotic cell death [30]. Our results demonstrated that ACE2 overexpression decreased TUNEL-positive cells, the activity of caspase3, the expression of NOX2 and P47, and increased bcl-2 expression and SOD activity. These results suggest that ACE2 may inhibit doxorubicin-induced cardiomyocyte apoptosis partially via inhibition of oxidative stress.

More recently, it was reported that AMP-activated protein kinase (AMPK) activation directly phosphorylates MAPK8, which mediates BCL2 phosphorylation, resulting in inhibition of cardiac apoptosis, and improvement in cardiac structure and function [31]. The inhibition of AMPK phosphorylation by doxorubicin was responsible for cardiomyocyte apoptosis and cardiac dysfunction [32]. Previous study found that ACE2 deficiency was associated with decreased phosphorylation of AMPK, increased cardiac lipotoxicity and myocardial insulin resistance, which worsened heart function [33]. In contrast, ACE2 activator ameliorates cardiac dysfunction in diabetic cardiomyopathy through increasing AMPK-α phosphorylation and activating PI3K/AKT pathway [34]. Activation of the PI3K/AKT pathway was involved in the protection against doxorubicin-induced apoptosis [35]. In this study, we found that ACE2 overexpression prohibited downregulation of AMPK and PI3K/AKT signaling pathways by doxorubicin, indicating that AMPK and PI3K/AKT signaling pathways activated by ACE2 overexpression may play an important cardioprotective effect on doxorubicin-induced cardiomyopathy.

Accumulating studies have demonstrated that infiltration of inflammatory cells was involved in doxorubicin-induced cardiac dysfunction [36]. Oxidative stress could directly induce inflammatory cytokines expression, which was significantly increased after doxorubicin injection [37]. ACE2 prevented AngII-induced inflammatory response [38]. Consistent with these findings, we found that the expressions of pro-inflammatory cytokines such as TNF-α, VACM-1 and ICAM-1 were markedly increased in doxorubicin-induced rats, and were markedly suppressed by ACE2 overexpression. ERK1/2, a member of mitogen-activated protein kinase family, are associated with activated inflammation [39]. Recent study also demonstrated that the expression levels of ERK1/2 upregulated by doxorubicin induced cell apoptosis [40]. Our results demonstrated that phosphorylation of ERK1/2 upregulated by doxorubicin was significantly suppressed by ACE2, indicating that ACE2 overexpression inhibits the inflammatory infiltration by inhibition of ERK1/2 expression.

Fibrosis plays a major role in adverse cardiac remodeling in doxorubicin-induced cardiomyopathy and post-MI myocardium [41]. In this study, ACE2 overexpression attenuated myocardial collagen deposition, improved LV remodeling and cardiac function, which were in line with the previous report [42]. Improvement in LV function was assessed by echocardiography, with improved LVEF and FS, coupled with reduction in LVESD and LVEDD by ACE2 overexpression. Cardiac fibrosis as a major contributor to end stage heart failure is affected by Matrix metalloproteinases(MMPs) and tissue inhibitor metalloproteinases(TIMPs), which results in degradation of extracellular matrix, collagen protein deposition, and cardiac dysfunction [43]. In this study, we found that ACE2 overexpression increased the MMP-9 expression, indicating that the collagen deposition inhibited by ACE2 overexpression is related to increased MMP-9 expression. Another crucial factor regulating collagen production in doxorubicin-induced cardiomyopathy is TGF-β1 [44]. TGF-β1, a protein secreted by cardiac myofibroblast and fibroblast, is involved in hypertrophy and fibrosis [45]. Our results showed that expression of TGF-β1 upregulated by doxorubicin was significantly decreased by ACE2, indicating that the improvement of cardiac fibrosis by ACE2 overexpression is associated with decrease of TGF-β1 expression.

ACE2 and ACE, a couple of enzymes with sequence homology and close functional relations, may regulate each other by feedback inhibition. Obviously, the balance between ACE and ACE2 is critical to the normal functional state of the RAS. In the present study, we found that ACE2 overexpression led to a shift in balance toward low ACE protein expression and hence a reduction in the concentration level of AngII. Furthermore, overexpression of ACE2 resulted in conversion of AngII to Ang (1–7). Previous studies demonstrated that direct delivery of Ang (1–7) into the myocardium had beneficial and independent cardiac protection effects in models of cardiac remodeling [12]. Ang (1–7) attenuated myocardial fibrosis and improved left ventricular remodeling and cardiac function in a rat model of diabetic cardiomyopathy [12, 46]. These results indicate that the effects of ACE2 overexpression on doxorubicin-induced cardiomyopathy may contribute to the net effect of decreased AngII and increased Ang (1–7) levels.

In this study, the therapeutic effects of ACE2 overexpression and cilazapril on doxorubicin-induced cardiomyopathy were first compared, and the results showed that treatment with cilazapril was able to reduce doxorubicin-induced cardiomyopathy, which are consistent with the cardioprotective effects of RAS blockade observed in models of doxorubicin-induced cardiomyopathy [47–49]. However, the degree of protection was significantly less than that observed following treatment with ACE2 overexpression. The difference may be due to that ACEI can inhibit AngII synthesis catalyzed by ACE in cardiac fibroblasts, but cannot inhibit AngII synthesis catalyzed by chymase in cardiac myocytes [50, 51]. Thus, ACEI may not completely prohibit RAS activation in doxorubicin-induced cardiomyopathy. However, ACE2 cleaves AngII into vasodilating and antiproliferative Ang (1–7). For these reasons, we could illustrate the more beneficial effects of ACE2 overexpression than ACEI on doxorubicin-induced cardiomyopathy in rats, which is consistent with the previous report [42].

Therapies with direct intramyocardial injection of ACE2 gene via adenovirus or lentivirus vectors have been studied in experimental heart failure [42, 52]. The direct intramyocardial injection can deliver genes selectively into target areas; however, this method induces mechanical damage and acute inflammatory response [53, 54], which may further cause the release of bio-markers of myocardial damage [17, 54]. Therefore, these side effects may become limitation of direct intramyocardial injection of gene therapies. Thus, looking for better treatment approaches of delivering ACE2 gene into heart need further studies.

In conclusion, our study demonstrates that ACE2 overexpression decreases the mortality of rats with doxorubicin-induced cardiomyopathy by decreasing apoptosis, inflammatory response, oxidative stress, myocardial fibrosis and improving left ventricular remodeling and cardiac function. And ACE2 overexpression is superior to cilazapril in protecting against doxorubicin-induced cardiomyopathy. The mechanisms underlying these therapeutic effects involve activation of the AMPK and PI3K-AKT pathways, inhibition of the ERK pathway, and decrease of TGF-β1 expression. In addition, these beneficial effects of ACE2 on doxorubicin-induced cardiomyopathy are likely to root in the interactions of shifting RAS components, such as decreased myocardium AngII levels, increased myocardium Ang (1–7) levels, and reduced ACE expression. Thus, ACE2 may be a novel therapeutic approach in the prevention and treatment of doxorubicin-induced cardiomyopathy.

## MATERIALS AND METHODS

### Preparation of ACE2 adenovirus vectors

The murine ACE2 cDNA was amplified by reverse-transcription polymerase chain reaction (RT-PCR) from RNA of a mouse kidney with the following primers: ACE2 F: 5′-GAAAGTTGCTCAGTGGATGGGAT-3′; ACE2 R: 5′- TTTGCTAAAAGGAAGTCTGAGCATC-3′. Recombinant adenoviruses (Ad) carrying the murine ACE2 (Ad-ACE2) or a control transgene EGFP (Ad-EGFP) were prepared with the AdMax system (Microbix Biosystems) according to our previously described experimental method [14].

### Animal models of doxorubicin-induced cardiomyopathy and gene treatment

190 male Wistar rats (8–10 weeks of age, 250–300 g body weight) were obtained from Shandong University Animal Center and housed in temperature-controlled cages with a 12-h light-dark cycle and given free access to water and regular diet. The cages were kept dry and clean. The animal protocol was reviewed and approved by the University of Shandong Institute Animal Care and Use Committee.

**Figure 10:**
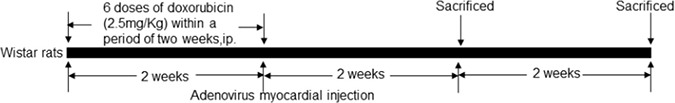
The protocols of animal experiments The protocols of adenovirus myocardial infection and doxorubicin administration in the Ad-EGFP and Ad-ACE2 groups.

All rats were randomly divided into treatment group (*n* = 170) and control group (*n* = 20). Doxorubicin (Sigma, Saint Louis, USA) was injected intraperitoneally (i.p.) via six equal doses (each containing 2.5 mg/kg Doxorubicin) within a period of two weeks, as previously described [55]. Age-matched rats injected with saline were used as control. Two weeks after the initial injection of doxorubicin, the remaining rats in the treatment group were randomly divided into Mock, Ad-EGFP, Ad-ACE2, and Cilazapril groups (*n* = 40 each group). As shown in Figure 10, rats in the Ad-EGFP and Ad-ACE2 groups were anesthetized with an intraperitoneal injection of 10% chloral hydrate (300 mg/kg) and mechanically ventilated with a VIP Bird ventilator (Bird Products Corp., Palm Spring, CA, USA) with a tidal volume, 3.0 ml and respiratory rate of 60 cycles/min. Anterior thoracotomy was performed under sterile conditions to open the pericardium and expose the heart. A total of 2 × 10^9^ pfu of Ad-EGFP and 2 × 10^9^ pfu of Ad-ACE2 in a final volume of 200 μl was delivered by a 30-gauge needle into six sites at depth of 1–2 mm in the left ventricular free wall as previously described [42]. The Mock group underwent the same surgical procedure but received an intramyocardial injection of normal saline only. The Cilazapril group was given cilazapril (Sigma, Saint Louis, USA) by intragastric intubation at a dose of 10 mg·kg^−1^·d^−1^.

Two weeks after adenovirus injection, eight rats from each group were euthanized for assessing the efficiency of Ad-ACE2 transfection, and the remaining rats in the treatment and control groups were euthanized 4 weeks after gene therapy. The myocardium of the left ventricle was collected for pathological and biochemical analysis.

### Blood pressure measurement

Heart rate, systolic blood pressure and diastolic blood pressure were measured in conscious rats using a noninvasive tail-cuff system with a special device designed for mice (Softron BP-98A. Tokyo, Japan). Blood pressures and heart rates were reported as mean of 3 consecutive measurements.

### Echocardiographic imaging

At the end of experiment, rats in the five groups underwent transthoracic echocardiographic scanning after anesthesia with intraperitoneal injection of 10% chloral hydrate (300 mg/kg), and two-dimensional echocardiography was performed with a 7.5-MHz phased-array transducer connected to a sector scanner (SONOS 7500, Philips Medical Systems, Andover, MA). The left ventricular end-diastolic diameter (LVEDD) and left ventricular end-systolic diameter (LVESD) were obtained from the parasternal long-axis view and the left ventricular fractional shortening was calculated according to the following formula: FS = (LVDd − LVDs)/LVDd × 100. Left ventricular ejection fraction (LVEF) was calculated according to the following formula: LVEF = (LVVd − LVVs)/LVVd × 100. All measurements were averaged for 3 consecutive cardiac cycles by an experienced technician who was blinded to study grouping.

### Real-time quantitative PCR

Total RNA was isolated from freshly isolated myocardial samples by use of Trizol reagent (Invitrogen) according to the manufacturer's protocol. The extracted mRNA was dissolved in RNase free water, and concentrations of the total RNA were detected using a spectrophotometer. One microgram of mRNA was used for reverse transcription using PrimeCriptTM RT reagent kit with gDNA Eraser (TaKaRa, Japan) containing a mixture of oligo and random primers. Amplification was performed using an iCycler iQ real-time PCR detection system. In the experiment, mRNA expression of β-actin and ACE2 was determined by means of SYBR Green technology (TaKaRa, Japan). β-actin was used as an internal control. Quantitative values were obtained from the threshold cycle value (Ct) and the 2^−∆∆CT^ method was used to determine relative gene expression levels. Primers for ACE2 and β-actin were as follows: ACE2, forward 5′-CGTATGGGTGAGTGATTTG-3′, reverse 5′-AGGAGGCTCGTAAGGTG-3′; β-actin, forward 5′-TGTTGCCCTAGACTTCGAGCA-3′, reverse 5′-GGACC CAGGAAGGAAGGCT-3.

### ACE2 activity assay

ACE2 activity was determined by use of assays based on internally quenched fluorescent substrates [Abz-Ser-Pro-3-nitro-Tyr-OH, M-2660 for ACE2 from Bachem, Torrance, CA] as described by Yan et al. [56] for ACE2 activity.

### Western blot analysis

Total proteins were extracted from myocardial tissues. Protein samples were separated on 10% SDS-PAGE and transferred to nitrocellulose membranes. After incubation in 5% skim milk for 1.5 h at room temperature, membranes were incubated overnight at 4°C with the primary antibodies (Table 2). After a wash and incubation with Specific conjugated peroxidase-labeled secondary antibodies, protein bands were visualized by use of the ECL reagents (Millipore Corp., MA, USA). GAPDH expression was used to ensure equal protein loading. Protein expression levels were determined by densitometry. All experiments were repeated for three times and the mean values derived.

**Table 2:** Antibodies used for western bloting

Antibody	Dilution	Cat.no.	Inc.
GAPDH	1:1000	2118S	Cell Signaling Technology, USA
ACE2	1:1000	ab108252	Abcam
collagen I	1:1000	ab34710	Abcam
collagen III	1:1000	ab7778	Abcam
ICAM-1	1:1000	ab53013	Abcam
VCAM-1	1:1000	ab134047	Abcam
TNF-α	1:1000	ab6671	Abcam
Nox2	1:500	sc-20782	Santa Cruz Biotechnology
p47	1:500	sc-14015	Santa Cruz Biotechnology
TGF-β1	1:1000	ab92486	Abcam
MMP-1	1:1000	ab52631	Abcam
MMP-9	1:1000	ab119906	Abcam
TIMP-1	1:1000	sc-5538	Santa Cruz Biotechnology
TIMP-2	1:1000	sc-5539	Santa Cruz Biotechnology
pERK	1:1000	4370S	Cell Signaling Technology, USA
ERK	1:1000	4695S	Cell Signaling Technology, USA
pAMPK	1:1000	2535L	Cell Signaling Technology, USA
AMPK	1:1000	5832S	Cell Signaling Technology, USA
c-caspase3	1:1000	ab2302	Abcam
caspase3	1:1000	ab13847	Abcam
Bcl-2	1:1000	ab32124	Abcam
iNOS	1:1000	ab15323	Abcam
pPI3K	1:1000	4228S	Cell Signaling Technology, USA
PI3K	1:1000	4257S	Cell Signaling Technology, USA
pAKT	1:1000	4051L	Cell Signaling Technology, USA
AKT	1:1000	4691S	Cell Signaling Technology, USA
ACE	1:1000	ab75762	Abcam

### TUNEL assay

TUNEL assay was performed using an Roche *in situ* cell death detection kit, POD (Mannheim, Germany) according to the manufacturer's instructions. Briefly, the sections were dewaxed and rehydrated using xylene and ethanol gradings, permeabilized using proteinase K and incubated with the TUNEL reaction mixture containing TdT and fluorescein labeled dUTP for 1 hour at 37°C. Sections were washed by PBS for 3 times and incubated with the converter-POD for 30mins at 37°C followed by DAB+ chromogen detection. After final washes, sections were counterstained with hematoxylin. Images were captured with a microscope (BX41, Olympus) and with digital camera (Spot Insight 2, Diagnostic Instruments, Inc.). For negative control, TdT was not included in the reaction mixture. The TUNEL-positive cells were quantified by randomly counting 12 different microscopic fields (magnification, 400×) for each section. Then the ratio of apoptotic cells was calculated by dividing the number of TUNEL-positive nuclei by the total number of counted nuclei.

### Histopathology and immunohistochemistry (IHC)

After being fixed in formalin and embedded in paraffin, tissue samples from the myocardium were cut into serial sections 4.5 μm thick and stained with hematoxylin-eosin. The sections were stained with Masson's trichrome to display the collagen components, which were quantitated by measuring the proportion of area positively stained with Masson's trichrome to the total left ventricular area in the section. Immunohistochemical staining with rabbit ACE2 antibody (Abcam), rabbit collagen I and III antibodies (Abcam) and rabbit VCAM-1 and TNF-α antibodies (Abcam) was used to determine the expression of ACE2, collagen I and III, VCAM-1 and TNF-α, respectively. All histopathological sections were viewed with a microscope (BX41, Olympus) and with digital camera (Spot Insight 2, Diagnostic Instruments, Inc.). Vessel areas were measured with Image Pro Plus software (Media Cybernetics Inc.).

### Measurements of SOD assay

The SOD activity in the heart was measured according to the method using a kit (NJBC, Nanjing, China). Tetrazolium salt can be made to form a red formazan dye by superoxide radicals generated by xanthine oxidase and hypoxanthine. The red formazan dye was measured and evaluated at the optical density of 550 nm by a spectrophotometer. The SOD activity was expressed as IU/mg protein.

### AngII and Ang (1–7) levels by ELISA

The concentration of AngII and Ang (1–7) in the rat myocardium and plasma was determined by using commercial enzyme-linked immunosorbent assay (ELISA) kits (Bachem, USA) following the manufacturer's recommendations [57]. In brief, the myocardium had been frozen and powdered in liquid nitrogen, the powdered tissue was mechanically homogenized with Iscove's culture medium containing a protease inhibitor cocktail (Sigma) on ice, using a homogenizer (Omni International Inc., Warrenton, VA, USA). Homogenized samples were centrifuged at 10,000 rpm for 10 min at 4°C. Each supernatant was then transferred into a fresh eppendorf-tube and stored at −80°C. Blood was obtained in a cocktail of protease inhibitors, and the levels of myocardial and plasma AngII and Ang (1–7) were determined by ELISA.

### Statistical analysis

Statistical analyses invovled use of SPSS 20 (SPSS Inc, Chicago, IL). Data were reported as mean ± SEM in bar graphs. Comparisons of parameters among more than two groups were analyzed by one-way ANOVA with least significant difference post-hoc analysis. Survival curves were plotted using Kaplan-Meier analysis (Log-Rank with Mantel-Coxtest). *p* value < 0.05 was considered significant.

## References

[1] Lefrak EA, Pitha J, Rosenheim S, Gottlieb JA (1973). A clinicopathologic analysis of adriamycin cardiotoxicity. Cancer.

[2] Minotti G, Menna P, Salvatorelli E, Cairo G, Gianni L (2004). Anthracyclines: molecular advances and pharmacologic developments in antitumor activity and cardiotoxicity. Pharmacol Rev.

[3] Sterba M, Popelova O, Vavrova A, Jirkovsky E, Kovarikova P, Gersl V, Simunek T (2013). Oxidative stress, redox signaling, and metal chelation in anthracycline cardiotoxicity and pharmacological cardioprotection. Antioxid Redox Signal.

[4] Takemura G, Fujiwara H (2007). Doxorubicin-induced cardiomyopathy from the cardiotoxic mechanisms to management. Prog Cardiovasc Dis.

[5] Ueno M, Kakinuma Y, Yuhki K, Murakoshi N, Iemitsu M, Miyauchi T, Yamaguchi I (2006). Doxorubicin induces apoptosis by activation of caspase-3 in cultured cardiomyocytes in vitro and rat cardiac ventricles in vivo. J Pharmacol Sci.

[6] Thandavarayan RA, Giridharan VV, Arumugam S, Suzuki K, Ko KM, Krishnamurthy P, Watanabe K, Konishi T, Schisandrin B (2015). prevents doxorubicin induced cardiac dysfunction by modulation of DNA damage, oxidative stress and inflammation through inhibition of MAPK/p53 signaling. PLoS One.

[7] Miyata S, Takemura G, Kosai K, Takahashi T, Esaki M, Li L, Kanamori H, Maruyama R, Goto K, Tsujimoto A, Takeyama T, Kawaguchi T, Ohno T (2010). Anti-Fas gene therapy prevents doxorubicin-induced acute cardiotoxicity through mechanisms independent of apoptosis. Am J Pathol.

[8] Lipshultz SE, Karnik R, Sambatakos P, Franco VI, Ross SW, Miller TL (2014). Anthracycline-related cardiotoxicity in childhood cancer survivors. Curr Opin Cardiol.

[9] Iarussi D, Indolfi P, Casale F, Coppolino P, Tedesco MA, Di Tullio MT (2001). Recent advances in the prevention of anthracycline cardiotoxicity in childhood. Curr Med Chem.

[10] Richard C, Lauzier B, Delemasure S, Talbot S, Ghibu S, Collin B, Senecal J, Menetrier F, Vergely C, Couture R, Rochette L (2008). Effects of angiotensin-1 converting enzyme inhibition on oxidative stress and bradykinin receptor expression during doxorubicin-induced cardiomyopathy in rats. J Cardiovasc Pharmacol.

[11] Wanless RB, Anand IS, Gurden J, Harris P, Poole-Wilson PA (1987). Regional blood flow and hemodynamics in the rabbit with adriamycin cardiomyopathy: effects of isosorbide dinitrate, dobutamine and captopril. J Pharmacol Exp Ther.

[12] Hao PP, Yang JM, Zhang MX, Zhang K, Chen YG, Zhang C, Zhang Y (2015). Angiotensin-(1–7) treatment mitigates right ventricular fibrosis as a distinctive feature of diabetic cardiomyopathy. Am J Physiol Heart Circ Physiol.

[13] Crackower MA, Sarao R, Oudit GY, Yagil C, Kozieradzki I, Scanga SE, Oliveira-dos-Santos AJ, da Costa J, Zhang L, Pei Y, Scholey J, Ferrario CM, Manoukian AS (2002). Angiotensin-converting enzyme 2 is an essential regulator of heart function. Nature.

[14] Zhao YX, Yin HQ, Yu QT, Qiao Y, Dai HY, Zhang MX, Zhang L, Liu YF, Wang LC, Liu DS, Deng BP, Zhang YH, Pan CM (2010). ACE2 overexpression ameliorates left ventricular remodeling and dysfunction in a rat model of myocardial infarction. Hum Gene Ther.

[15] Patel VB, Zhong JC, Grant MB, Oudit GY (2016). Role of the ACE2/Angiotensin 1–7 Axis of the Renin-Angiotensin System in Heart Failure. Circ Res.

[16] Vickers C, Hales P, Kaushik V, Dick L, Gavin J, Tang J, Godbout K, Parsons T, Baronas E, Hsieh F, Acton S, Patane M, Nichols A (2002). Hydrolysis of biological peptides by human angiotensin-converting enzyme-related carboxypeptidase. J Biol Chem.

[17] Baldazzi F, Jorgensen E, Ripa RS, Kastrup J (2008). Release of biomarkers of myocardial damage after direct intramyocardial injection of genes and stem cells via the percutaneous transluminal route. Eur Heart J.

[18] Hamed S, Barshack I, Luboshits G, Wexler D, Deutsch V, Keren G, George J (2006). Erythropoietin improves myocardial performance in doxorubicin-induced cardiomyopathy. Eur Heart J.

[19] Wang S, Zhang M, Liang B, Xu J, Xie Z, Liu C, Viollet B, Yan D, Zou MH (2010). AMPKalpha2 deletion causes aberrant expression and activation of NAD(P)H oxidase and consequent endothelial dysfunction in vivo: role of 26S proteasomes. Circ Res.

[20] Wang S, Zhang C, Zhang M, Liang B, Zhu H, Lee J, Viollet B, Xia L, Zhang Y, Zou MH (2012). Activation of AMP-activated protein kinase alpha2 by nicotine instigates formation of abdominal aortic aneurysms in mice in vivo. Nat Med.

[21] Zhou L, Chen L, Wang J, Deng Y (2015). Astragalus polysaccharide improves cardiac function in doxorubicin-induced cardiomyopathy through ROS-p38 signaling. Int J Clin Exp Med.

[22] Chen PY, Hou CW, Shibu MA, Day CH, Pai P, Liu ZR, Lin TY, Viswanadha VP, Kuo CH, Huang CY (2017). Protective effect of Co-enzyme Q10 On doxorubicin-induced cardiomyopathy of rat hearts. Environ Toxicol.

[23] Singal PK, Li T, Kumar D, Danelisen I, Iliskovic N (2000). Adriamycin-induced heart failure: mechanism and modulation. Mol Cell Biochem.

[24] Aldieri E, Bergandi L, Riganti C, Costamagna C, Bosia A, Ghigo D (2002). Doxorubicin induces an increase of nitric oxide synthesis in rat cardiac cells that is inhibited by iron supplementation. Toxicol Appl Pharmacol.

[25] Yu B, Meng F, Yang Y, Liu D, Shi K (2016). NOX2 Antisense Attenuates Hypoxia-Induced Oxidative Stress and Apoptosis in Cardiomyocyte. Int J Med Sci.

[26] Berasi SP, Xiu M, Yee AS, Paulson KE (2004). HBP1 repression of the p47phox gene: cell cycle regulation via the NADPH oxidase. Mol Cell Biol.

[27] Dong Q, Chen L, Lu Q, Sharma S, Li L, Morimoto S, Wang G (2014). Quercetin attenuates doxorubicin cardiotoxicity by modulating Bmi-1 expression. Br J Pharmacol.

[28] Chen CT, Wang ZH, Hsu CC, Lin HH, Chen JH (2015). *In Vivo* Protective Effects of Diosgenin against Doxorubicin-Induced Cardiotoxicity. Nutrients.

[29] Qi YF, Zhang J, Wang L, Shenoy V, Krause E, Oh SP, Pepine CJ, Katovich MJ, Raizada MK (2016). Angiotensin-converting enzyme 2 inhibits high-mobility group box 1 and attenuates cardiac dysfunction post-myocardial ischemia. J Mol Med (Berl).

[30] Patel VB, Zhong JC, Fan D, Basu R, Morton JS, Parajuli N, McMurtry MS, Davidge ST, Kassiri Z, Oudit GY (2014). Angiotensin-converting enzyme 2 is a critical determinant of angiotensin II-induced loss of vascular smooth muscle cells and adverse vascular remodeling. Hypertension.

[31] Zou MH, Xie Z (2013). Regulation of interplay between autophagy and apoptosis in the diabetic heart: new role of AMPK. Autophagy.

[32] Gratia S, Kay L, Potenza L, Seffouh A, Novel-Chate V, Schnebelen C, Sestili P, Schlattner U, Tokarska-Schlattner M (2012). Inhibition of AMPK signalling by doxorubicin: at the crossroads of the cardiac responses to energetic, oxidative, and genotoxic stress. Cardiovasc Res.

[33] Patel VB, Mori J, McLean BA, Basu R, Das SK, Ramprasath T, Parajuli N, Penninger JM, Grant MB, Lopaschuk GD, Oudit GY (2016). ACE2 Deficiency Worsens Epicardial Adipose Tissue Inflammation and Cardiac Dysfunction in Response to Diet-Induced Obesity. Diabetes.

[34] Murca TM, Moraes PL, Capuruco CA, Santos SH, Melo MB, Santos RA, Shenoy V, Katovich MJ, Raizada MK, Ferreira AJ (2012). Oral administration of an angiotensin-converting enzyme 2 activator ameliorates diabetes-induced cardiac dysfunction. Regul Pept.

[35] Ren D, Zhu Q, Li J, Ha T, Wang X, Li Y (2012). Overexpression of angiopoietin-1 reduces doxorubicin-induced apoptosis in cardiomyocytes. J Biomed Res.

[36] Li L, Takemura G, Li Y, Miyata S, Esaki M, Okada H, Kanamori H, Khai NC, Maruyama R, Ogino A, Minatoguchi S, Fujiwara T, Fujiwara H (2006). Preventive effect of erythropoietin on cardiac dysfunction in doxorubicin-induced cardiomyopathy. Circulation.

[37] Wang L, Zhang TP, Zhang Y, Bi HL, Guan XM, Wang HX, Wang X, Du J, Xia YL, Li HH (2016). Protection against doxorubicin-induced myocardial dysfunction in mice by cardiac-specific expression of carboxyl terminus of hsp70-interacting protein. Sci Rep.

[38] Zhong J, Guo D, Chen CB, Wang W, Schuster M, Loibner H, Penninger JM, Scholey JW, Kassiri Z, Oudit GY (2011). Prevention of angiotensin II-mediated renal oxidative stress, inflammation, and fibrosis by angiotensin-converting enzyme 2. Hypertension.

[39] Son Y, Cheong YK, Kim NH, Chung HT, Kang DG, Pae HO (2011). Mitogen-Activated Protein Kinases and Reactive Oxygen Species: How Can ROS Activate MAPK Pathways? J Signal Transduct.

[40] Singla DK, Ahmed A, Singla R, Yan B (2012). Embryonic stem cells improve cardiac function in Doxorubicin-induced cardiomyopathy mediated through multiple mechanisms. Cell Transplant.

[41] Merino H, Singla DK (2014). Notch-1 mediated cardiac protection following embryonic and induced pluripotent stem cell transplantation in doxorubicin-induced heart failure. PLoS One.

[42] Dong B, Yu QT, Dai HY, Gao YY, Zhou ZL, Zhang L, Jiang H, Gao F, Li SY, Zhang YH, Bian HJ, Liu CX, Wang N (2012). Angiotensin-converting enzyme-2 overexpression improves left ventricular remodeling and function in a rat model of diabetic cardiomyopathy. J Am Coll Cardiol.

[43] Jugdutt BI, Menon V, Kumar D, Idikio H (2002). Vascular remodeling during healing after myocardial infarction in the dog model: effects of reperfusion, amlodipine and enalapril. J Am Coll Cardiol.

[44] Ma S, Li X, Dong L, Zhu J, Zhang H, Jia Y (2016). Protective effect of Sheng-Mai Yin, a traditional Chinese preparation, against doxorubicin-induced cardiac toxicity in rats. BMC Complement Altern Med.

[45] Wang B, Hao J, Jones SC, Yee MS, Roth JC, Dixon IM (2002). Decreased Smad 7 expression contributes to cardiac fibrosis in the infarcted rat heart. Am J Physiol Heart Circ Physiol.

[46] Hao P, Yang J, Liu Y, Zhang M, Zhang K, Gao F, Chen Y, Zhang C, Zhang Y (2015). Combination of angiotensin-(1–7) with perindopril is better than single therapy in ameliorating diabetic cardiomyopathy. Sci Rep.

[47] Ibrahim MA, Ashour OM, Ibrahim YF, El-Bitar HI, Gomaa W, Abdel-Rahim SR (2009). Angiotensin-converting enzyme inhibition and angiotensin AT(1)-receptor antagonism equally improve doxorubicin-induced cardiotoxicity and nephrotoxicity. Pharmacol Res.

[48] Arozal W, Watanabe K, Veeraveedu PT, Thandavarayan RA, Harima M, Sukumaran V, Suzuki K, Kodama M, Aizawa Y (2010). Effect of telmisartan in limiting the cardiotoxic effect of daunorubicin in rats. J Pharm Pharmacol.

[49] Soga M, Kamal FA, Watanabe K, Ma M, Palaniyandi S, Prakash P, Veeraveedu P, Mito S, Kunisaki M, Tachikawa H, Kodama M, Aizawa Y (2006). Effects of angiotensin II receptor blocker (candesartan) in daunorubicin-induced cardiomyopathic rats. Int J Cardiol.

[50] Singh VP, Baker KM, Kumar R (2008). Activation of the intracellular renin-angiotensin system in cardiac fibroblasts by high glucose: role in extracellular matrix production. Am J Physiol Heart Circ Physiol.

[51] Singh VP, Le B Khode R, Baker KM, Kumar R (2008). Intracellular angiotensin II production in diabetic rats is correlated with cardiomyocyte apoptosis, oxidative stress, and cardiac fibrosis. Diabetes.

[52] Wang YK, Shen D, Hao Q, Yu Q, Wu ZT, Deng Y, Chen YF, Yuan WJ, Hu QK, Su DF, Wang WZ (2014). Overexpression of angiotensin-converting enzyme 2 attenuates tonically active glutamatergic input to the rostral ventrolateral medulla in hypertensive rats. Am J Physiol Heart Circ Physiol.

[53] Kamihata H, Matsubara H, Nishiue T, Fujiyama S, Tsutsumi Y, Ozono R, Masaki H, Mori Y, Iba O, Tateishi E, Kosaki A, Shintani S, Murohara T (2001). Implantation of bone marrow mononuclear cells into ischemic myocardium enhances collateral perfusion and regional function via side supply of angioblasts, angiogenic ligands, and cytokines. Circulation.

[54] Suzuki K, Murtuza B, Beauchamp JR, Brand NJ, Barton PJ, Varela-Carver A, Fukushima S, Coppen SR, Partridge TA, Yacoub MH (2004). Role of interleukin-1beta in acute inflammation and graft death after cell transplantation to the heart. Circulation.

[55] Siveski-Iliskovic N, Kaul N, Singal PK (1994). Probucol promotes endogenous antioxidants and provides protection against adriamycin-induced cardiomyopathy in rats. Circulation.

[56] Yan ZH, Ren KJ, Wang Y, Chen S, Brock TA, Rege AA (2003). Development of intramolecularly quenched fluorescent peptides as substrates of angiotensin-converting enzyme 2. Anal Biochem.

[57] Fukada M, Kato S, Miyoshi M, Yamaguchi K, Imoto T, Watanabe T (2005). Systemic administration of lipopolysaccharide upregulates angiotensin II expression in rat renal tubules: immunohistochemical and ELISA studies. Peptides.

